# Predictive factors for survival in borderline resectable and locally advanced pancreatic cancer: are these really two different entities?

**DOI:** 10.1186/s12893-023-02200-6

**Published:** 2023-09-30

**Authors:** Luís Filipe Abreu de Carvalho, Filip Gryspeerdt, Niki Rashidian, Kobe Van Hove, Lambertine Maertens, Suzane Ribeiro, Anne Hoorens, Frederik Berrevoet

**Affiliations:** 1 Department of HPB surgery and liver transplantation, Ghent University Hospital, Corneel Heymanslaan 10, 9000 Ghent, Belgium; 2 Department of Gastroenterology, Division of Digestive Oncology, Ghent University Hospital, Ghent, Belgium; 3 Department of Pathology, Ghent University Hospital, Ghent, Belgium

**Keywords:** Pancreatic cancer, Borderline resectable pancreatic cancer, Locally advanced pancreatic cancer, Neoadjuvant chemotherapy, FOLFIRINOX

## Abstract

**Background:**

The treatment of borderline resectable (BR) and locally advanced (LA) pancreatic ductal adenocarcinoma (PDAC) has evolved with a wider application of neoadjuvant chemotherapy (NACHT). The aim of this study was to identify predictive factors for survival in BR and LA PDAC.

**Methods:**

Clinicopathologic data of patients with BR and LA PDAC who underwent surgical exploration between January 2011 and June 2021 were retrospectively collected. Survival from the date of surgery was estimated using the Kaplan-Meier method. Simple and multiple Cox proportional hazards models were fitted to identify factors associated with survival. Surgical resection was analyzed in combination with the involvement of lymph nodes as this last was only known after a formal resection.

**Results:**

Ninety patients were surgically explored (BR: 45, LA: 45), of which 51 (57%) were resected (BR: 31, LA: 20). NACHT was administered to 43 patients with FOLFIRINOX being the most frequent regimen applied (33/43, 77%). Major complications (Clavien-Dindo grade III and IV) occurred in 7.8% of patients and 90-day mortality rate was 3.3%. The median overall survival since surgery was 16 months (95% CI 12-20) in the group which underwent surgical resection and 10 months (95% CI 7-13) in the group with an unresectable tumor (*p=*0.001). Cox proportional hazards models showed significantly lower mortality hazard for surgical resection compared to no surgical resection, even after adjusting for National Comprehensive Cancer Network  (NCCN) classification and administration of NACHT [surgical resection with involved lymph nodes vs no surgical resection (cHR 0.49; 95% CI 0.29-0.82; *p=*0.007)]. There was no significant difference in survival between patients with BR and LA disease (cHR= 1.01; 95% CI 0.63-1.62; *p=*0.98).

**Conclusions:**

Surgical resection is the only predictor of survival in patients with BR and LA PDAC, regardless of their initial classification as BR or LA. Our results suggest that surgery should not be denied to patients with LA PDAC a priori. Prospective studies including patients from the moment of diagnosis are required to identify biologic and molecular markers which may allow a better selection of patients who will benefit from surgery.

**Supplementary Information:**

The online version contains supplementary material available at 10.1186/s12893-023-02200-6.

## Background

Pancreatic cancer has an ominous prognosis with a 5-year survival of 9% [1]. At the time of diagnosis, 50-60% of patients present with metastases, which precludes the possibility of a curative surgical approach. Surgery is possible in only 10-20% of patients. In the other 30-40% of the patients, the probability of a complete surgical resection is uncertain due to the vascular relation of the tumor, which is nowadays defined as borderline resectable (BR) pancreatic ductal adenocarcinoma (PDAC) or locally advanced (LA) PDAC according to the National Comprehensive Cancer Network (NCCN) guidelines [2–4]. Surgical resection is stated as the only possibility of cure, but it is the synergic combination of surgery with adjuvant systemic therapy which drastically improved overall survival (OS) [5]. Currently, FOLFIRINOX is the preferred adjuvant therapy [6]. After initially showing a benefit in the metastasized setting [7], it is now also applied as neoadjuvant chemotherapy (NACHT) in patients with BR and LA disease [8]. Vascular conflict in BR tumors is typically less extensive, which might allow for upfront surgical resection with a more extensive surgical procedure. Nowadays, NACHT is also recommended for BR [9]. In contrast, LA tumors generally undergo NACHT in an attempt to downsize the tumor to resectable disease [2]. However, the evaluation of response to NACHT, whether resorting to iconographic response evaluation criteria in solid tumors (RECIST) or monitoring a decrease of the tumor marker CA 19.9, lacks the specificity needed to predict surgical resectability. As a result, it necessitates surgical exploration for patients who show no evident progression and maintain a favorable performance status [2, 10, 11]. Within this context, the question arises as whether BR and LA should be considered as distinct entities, defined by specific anatomical boundaries with differing prognoses, or if they should be viewed as a continuum of the same advanced disease. Therefore, the aim of this study is to identify predictive factors for survival in BR and LA PDAC at a high-volume tertiary center.

## Methods

### Patient selection and collection of data

All consecutive patients who underwent a surgical exploration for BR or LA at our hospital between January 2011 and June 2021 were identified, based on the review of clinical files and surgical records. At diagnosis all patients underwent a triphasic contrast-enhanced computed tomography (CT), occasionally with the addition of magnetic resonance imaging (MRI). Diagnostic work-up included a chest CT and baseline CA 19.9. The tumors were classified as resectable, BR or LA according to the NCCN guidelines [3]. In summary, a tumor was considered BR when there was contact with the superior mesenteric artery (SMA) to an extent inferior to 180° or with the common hepatic artery without extension to the coeliac axis. When tumor contact with the superior mesenteric vein (SMV) or portal vein was wider than 180° or venous thrombosis was present, the tumor remained classified as BR, if segmental resection and venous reconstruction remained feasible. A tumor was classified as LA when there was solid tumor contact with SMA wider than 180°, extension to the coeliac axis or unreconstructible involvement of the SMV or portal vein. Evaluation of iconographic response after NACHT was performed according to the RECIST guidelines [11]. All patients were discussed at a multidisciplinary oncology meeting, with extensive review of all imaging by radiologists and surgeons. The NCCN classification was determined and the indication for upfront surgery or NACHT was agreed upon. For patients who had multiple cycles of NACHT with interval evaluations, only the last assessment before surgery was considered for this study. A biopsy confirming the diagnosis of PDAC was required before start of NACHT. The data collected included demographics, details on the surgical procedure and postoperative period, morbidity during index admission according to Clavien-Dindo classification (minor complications classified as grade I and II, major complications corresponding to grade III or IV), 90-day mortality and oncologic follow-up [12].

### Study endpoints

The primary objective of the study was to identify factors associated with survival since surgery. Several factors that could potentially influence survival outcomes in patients with PDAC were investigated including surgical resection, lymph node involvement, use of NACHT, chemotherapy regimen, RECIST and NCCN classification. Secondary objectives were the assessment of OS, surgical resectability and postoperative outcomes such as post operative morbidity and length of hospital stay.

### Chemotherapy, surgical procedure, and follow-up

NACHT with FOLFIRINOX or other scheme were administrated according to standard of care and response was evaluated every 2 months with triphasic CT abdomen, CT chest, CA 19-9 and occasionally MRI abdomen. The value of CA19-9 was considered normal if inferior to 37 kU/L. In the absence of iconographic progression, and decreased or stable CA19.9 after NACHT in patients with good performance status, surgical exploration was performed to evaluate resectability. Surgical exploration commenced with exclusion of peritoneal and liver metastases, followed by evaluation of local resectability. When fibrous tissue was present in contact with a venous vessel, a vascular resection was preferred. When fibrous tissue was in contact with de SMA or common hepatic artery, periadventitial dissection was performed at the blood vessel with arterial divestment and the hard tissue was sampled for frozen section analyses [13]. Dissection only continued if frozen section analysis showed no malignancy. The finding of arterial tumoral invasion at a later point of the dissection, after committing to resection and achieving a point of no return, was considered a R2 resection. During the surgical exploration, tumors requiring an arterial resection and reconstruction to obtain a complete tumoral excision were considered unresectable. Arterial resection was only considered in case of left pancreatectomy with coeliac axis resection [14]. Venous invasion was not a limitation for resection if a reconstruction was deemed possible. Pancreatic resections were performed according to the tumoral location with pancreaticoduodenectomy, left pancreatectomy or total pancreatectomy with associated lymphadenectomy. The department of hepatopancreatobiliary surgery of Ghent University Hospital is considered a high-volume referral center for pancreatic surgery. Pancreatic specific postoperative complications were analyzed according to the definitions of the international study group of pancreatic surgery and only the clinically relevant pancreatic fistula type B and C were considered [15–17]. Follow-up occurred according to the guidance of the oncologist in charge following the existent guidelines at the referral center, after postoperative surgical consultation. The first CT was performed within 3-6 weeks after the surgery, before starting adjuvant chemotherapy. When neoadjuvant chemotherapy was administered, the adjuvant therapy was given until completion of 12 cycles. In case of R1 or R2 resection, radiotherapy was advised after completion of adjuvant chemotherapy. Standardized macroscopic histopathological assessment of pancreatic resection specimens was performed according to the Leeds protocol in all specimens with evaluation of transection margins and dissection surfaces [18, 19]. The pancreatic and biliary transection margins were analyzed with frozen section. The circumferential resection margins were carefully analyzed. A margin was considered positive if the tumor was at or within 1 mm (≤1 mm) of the margin (R1). Tumor response after NACHT was scored in resected pancreatic specimens according to the grading system of College of American Pathologists (0: No viable cancer cells; 1: Single cells or rare small groups of cancer cells; 2: Residual cancer with evident tumor regression, but more than single cells or rare small groups of cancer cells; 3: Extensive residual cancer with no evident tumor regression) [20]. Lymph node ratio (LNR) was calculated in the patients who underwent a surgical resection by dividing the number of invaded lymph nodes by the number of retrieved lymph nodes and categorized as low LNR (0 – 0.2) or high LNR (>0.2) [21].

### Statistical analysis

Continuous variables were described with median and interquartile range (IQR) and they were compared between groups using student’s t-test in case of a normal distribution, otherwise Mann-Whitney U test was applied. Categorical data were described with counts and percentages. Chi-square test or Fisher’s exact test was used to compare categorical variables. Survival from the date of surgery was estimated using the Kaplan-Meier method. The log-rank test was used to compare survival curves between groups. Both simple and multiple Cox proportional hazards models were fitted to identify preoperative factors associated with survival. Surgical resection and NACHT were respectively analyzed in conjugation with lymph node involvement and RECIST instead of being considered separately as dichotomic variables because of their dependence relation. Lymph node status is only known in patients who had a formal resection and RECIST is only determined for patients who received NACHT. This allowed a better fitting of the proportional hazards models. Hazard ratios (HRs) from simple models and conditional hazard ratios (cHRs) from the multiple model were reported with 95% Wald confidence interval (CI) and Wald *p*-value. All statistical analyses were performed with IBM® SPSS® statistics 28.0 (2020, Armonk, New York, United States) and open-source R-software [22]. A two-sided *P*-value of < 0.05 was considered statistically significant.

## Results

A total of 570 patients underwent a pancreatic resection between January 2011 and June 2021. After exclusion of benign pathologies, cystic lesions, malignancies other than PDAC, and patients with upfront resectable PDAC, 90 patients with BR or LA PDAC were included in this analysis. This cohort includes only patients who underwent surgery, it is not representative for those with obvious disease progression or metastases. The characteristics of the study population are depicted in Table 1. There were no significant demographic differences between the two groups. Pancreatic head was the most common tumor location, found in a total of 69 patients (76.7%) and pancreaticoduodenectomy was the most common surgical procedure, performed in 43 patients (47.8%). In 48% (43/90) of the study population, NACHT was administered and 77% (33/43) of these patients had FOLFIRINOX while 23% (10/43) received other regimens. In the FOLFIRINOX group 64% (21/33) of the patients underwent a resection. R0 resection rate in this group was 21.2% (7/33), R1 resection rate was 42.4% (14/33) and 36.4% (12/33) of the patients had an unresectable tumor. The patients who received other regimens showed a 60% (6/10) resection rate, R1 resection rate was 50% (5/10), R2 resection rate was 10 % (1/10), there were no R0 resections and 40% (4/10) of the tumors were unresectable. The group of LA received NACHT more frequently [27/45 (62.8%) vs 16/45 (35.6%), *p=*0.034], with FOLFIRINOX being the most frequent regimen applied (33/43, 77%). Partial response according to RECIST with decrease of CA 19.9 was observed in 72% of the patients who received NACHT (31/43), without a significant difference between BR and LA [11/18 (68.8%) vs 20/27 (72.1%), *p=*0.737]. One patient with LA had an increase of CA 19.9 after NACHT (184 273 kU/L), all other patients who received NACHT had a decreased or stable CA 19.9. Fifty-one patients (57%) had a surgical resection (Table 2). R0 resection was achieved in 6 (13.6%) patients with BR and 5 (11.1%) patients with LA, while R1 resection was achieved in 24 (54.5%) patients with BR and 14 (31.1%) patients with LA (*p=*0.075) (Table 1). Fourteen patients with BR (29.5%) and 25 patients with LA (55.6%) (*p=*0.075) were deemed unresectable at the time of exploration, with vascular infiltration being the most common reason in both groups [10/14 (76%) vs 21/25 (88%), *p=*0.249]. There were less retrieved [17 (13-29) vs 22 (11-30), *p=*0.609] and involved [1 (0-2) vs 3 (0-6), *p=*0.024] lymph nodes in patients with LA compared to patients with BR. Except for this finding the other results were alike (Table 2). Major complications occurred in a total of 7 patients (7.8%) and 90-day mortality rate was 3.3 % (3 patients) (Table 1). Delayed gastric emptying was the most common complication occurring in 33% of the resected patients (17/51) and clinically relevant pancreatic fistula rate was 10% (5/51) (Table 2). After surgical resection, 81.8% of the patients (36/51) received adjuvant chemotherapy, this rate did not differ significantly between BR and LA (*p=*0.690).

**Table 1:** Patient characteristics

**Characteristic**	**BR (*n =* 45)**	**LA (*n =* 45)**	**Total (*n =* 90)**	***p*-value**
**Gender, n (%)**				
Female	16 (35.6)	23 (51.1)	39 (43.3)	0.202
Male	29 (64.4)	22 (48.9)	51 (56.7)	
**Age, (years) median (IQR)**	66 (58 - 73)	70 (60 - 73)	69 (59 -73)	0.678
**BMI, (kg/m2) median (IQR)**	25 (22 - 27)	24 (21 - 27)	24 (21 - 27)	0.190
**Tumor location, n (%)**				
Pancreatic head	38 (84.4)	31 (68.9)	69 (76.7)	0.235
Pancreatic body	6 (13.4)	12 (26.7)	18 (20)	
Pancreatic tail	1 (2.2)	2 (4.4)	3 (3.3)	
**NACHT, n (%)**	16 (35.6)	27 (62.8)	43 (47.8)	0.034
**Regimen**				
Folfirinox	12 (75)	21 (77.8)	33 (76.7)	0.870
Gemcitabine based	2 (12.5)	4 (14.8)	6 (14)	
Other	2 (12.5)	2 (7.4)	4 (9.3)	
**Nr cycles, median (IQR)**	5 (4 - 8)	6 (4 – 8)	5 (4-8)	0.476
**CA 19-9, (kU/L) median (IQR)**				
Upfront surgery	249 (22 – 6710)	52 (23 – 255)	193 (23 – 873)	0.264
Pre-NACHT	85 (26 – 232)	190 (85 - 543)	141 (52 – 520)	0.138
Post-NACHT	40 (4 – 91)	41 (16 – 158)	41 (13 -120)	0.472
**RECIST, n (%)**				
Partial response	11 (68.8)	20 (74.1)	31 (72.1)	0.737
Stable disease	5 (31.2)	7 (25.9)	12 (27.9)	
**Surgical Procedure, n (%)**				
Duodenopancreatectomy	26 (57.8)	17 (37.8)	43 (47.8)	0.023
Left pancreatectomy	2 (4.4)	3 (6.7)	5 (5.6)	
Total pancreatectomy	3 (6.7)	0 (0)	3 (3.3)	
Exploration/derivation	14 (31.1)	25 (55.5)	39 (43.3)	
**Venous resection, n (%)**	10 (23)	2 (5)	12 (15)	0.029
Wedge resection	2 (20)	1 (50)	3 (25)	0.455
Segment resection	8 (80)	1 (50)	9 (75)	
**Resectability, n (%)**				
R0	6 (13.6)	5 (11.1)	11 (12.4)	0.075
R1	24 (54.5)	14 (31.1)	38 (42.4)	
R2	1 (2.3)	1 (2.2)	2 (2.2)	
Unresectable	14 (29.5)	25 (55.6)	39 (43)	
Vascular infiltration	10 (76)	21 (88)	31 (85)	0.249
Liver metastases	1 (8)	3 (12)	4 (10)	
Peritoneal metastases	2 (16)	0 (0)	2 (5)	
**Clavien-Dindo, n (%)**				
Minor complication (Grade I-II)	12 (26.7)	18 (40)	30 (33.3)	0.263
Major complication (Grade III-IV)	2 (4.4)	5 (11.1)	7 (7.8)	0.434
**90-day mortality, n (%)**	3 (6.7)	0 (0)	3 (3.3)	0.242

*BR* Borderline resectable pancreatic ductal adenocarcinoma, *LA* Locally advanced pancreatic ductal adenocarcinoma, *NA* Not applicable, *IQR* Interquartile range, *BMI* Body Mass Index, *NACHT* Neoadjuvant chemotherapy, *RECIST* Response evaluation criteria in solid tumors

**Table 2:** Patients with surgical resection

	**BR (*n =* 31)**	**LA (*n =* 20)**	**Total (*n =* 51)**	***p*-value**
**T stage, n (%)**				
pT1	1 (3)	4 (20)	5 (9.8)	0.341
pT2	10 (32)	5 (25)	15 (29.4)	
pT3	13 (42)	7 (35)	20 (39.2)	
pT4	7 (23)	4 (20)	11 (21.6)	
**N stage, n (%)**				
pN0	7 (23)	7 (35)	14 (27.5)	0.140
pN1	16 (51)	12 (60)	28 (54.9)	
pN2	8 (26)	1 (5)	9 (17.6)	
**Retrieved lymph nodes, median (IQR)**	22 (11 - 30)	17 (13 – 29)	18 (12 - 29)	0.609
**Involved lymph nodes, median (IQR)**	3 (0 - 6)	1 (0 - 2)	2 (0 - 4)	0.024
**Duration of surgery, (min) median (IQR**)	480 (420 -540)	451 (411-523)	475 (415-525)	0.576
**Pancreatic specific complications, n (%)**				
Delayed gastric emptying	10 (32.2)	7 (35)	17 (33.3)	0.150
Pancreatic fistula	1 (3.2)	4 (20)	5 (10)	0.116
Hemorrhage	4 (12.9)	2 (10)	6 (11.7)	0.886
Bile leakage	1 (3.2)	1 (5)	2 (3.9)	1
**Hospital stay, (days) median (IQR)**	12 (10 - 16)	13 (10 - 14)	12 (10 -14)	0.968
**Adjuvant chemotherapy, n (%)**	23 (85.2)	13 (76.5)	36 (81.8)	0.690
**Time from surgery until start adjuvant chemotherapy, (days) median (IQR)**	64 (40 - 303)	60 (48 - 94)	60 (48 - 98)	0.657

*BR* Borderline resectable pancreatic ductal adenocarcinoma, *LA* Locally advanced pancreatic ductal adenocarcinoma, *NA* Not applicable, *IQR* Interquartile range

**Fig. 1:**
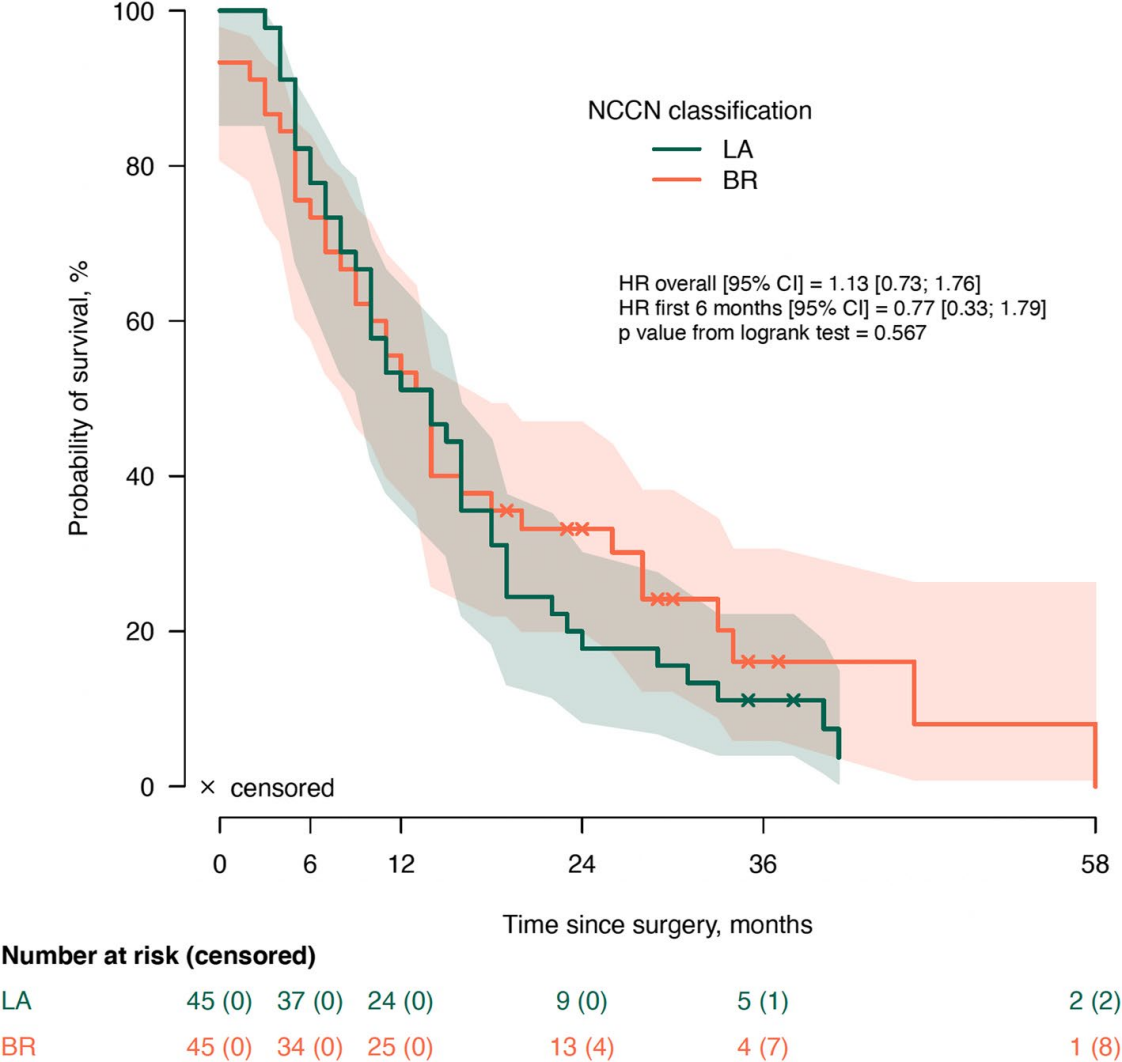
Overall survival according to NCCN classification. NCCN: National Comprehensive Cancer Network; LA: Locally advanced pancreatic ductal adenocarcinoma; BR: borderline resectable pancreatic ductal adenocarcinoma

**Fig. 2:**
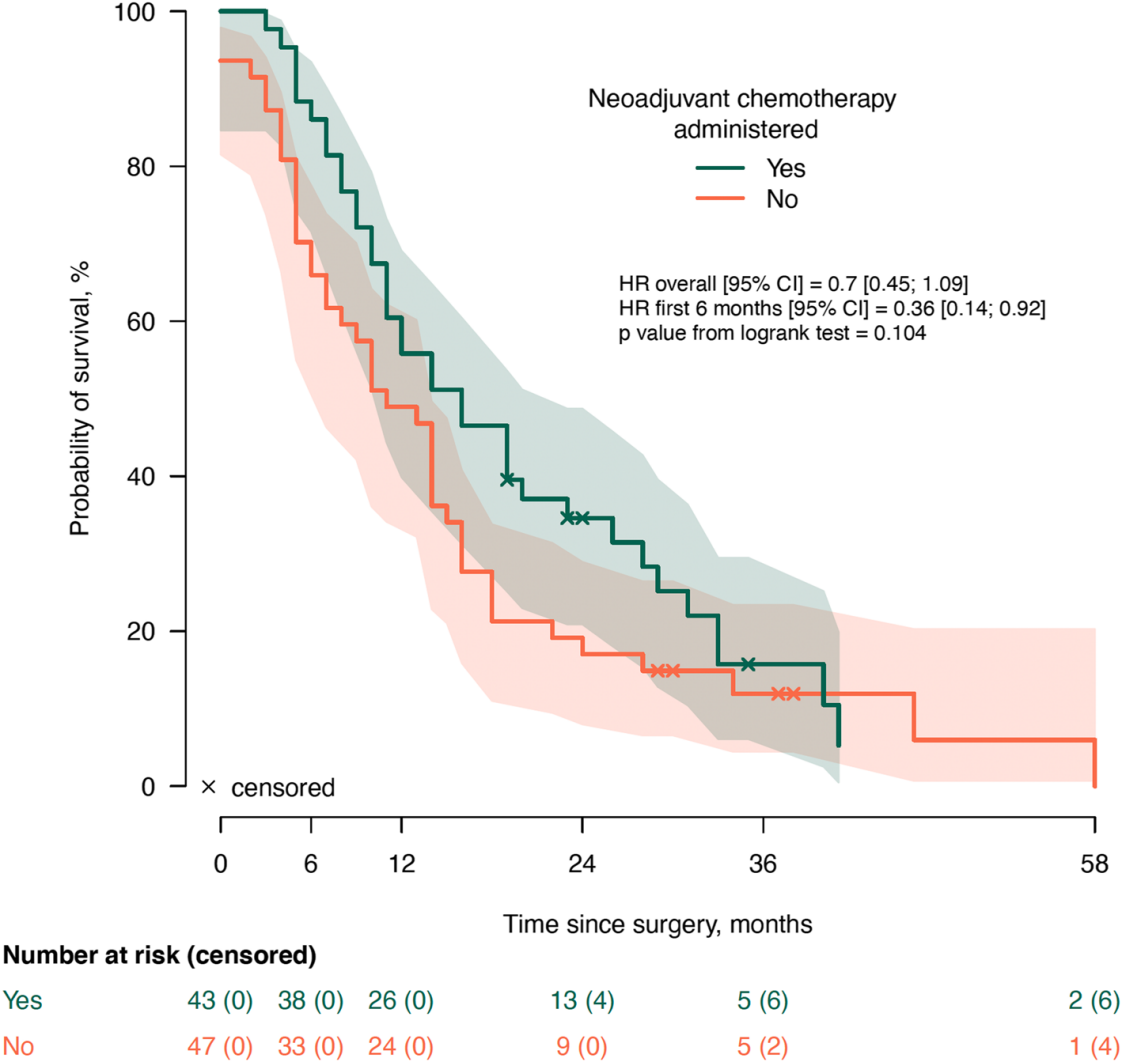
Overall survival according to the administration of neoadjuvant chemotherapy

**Fig. 3:**
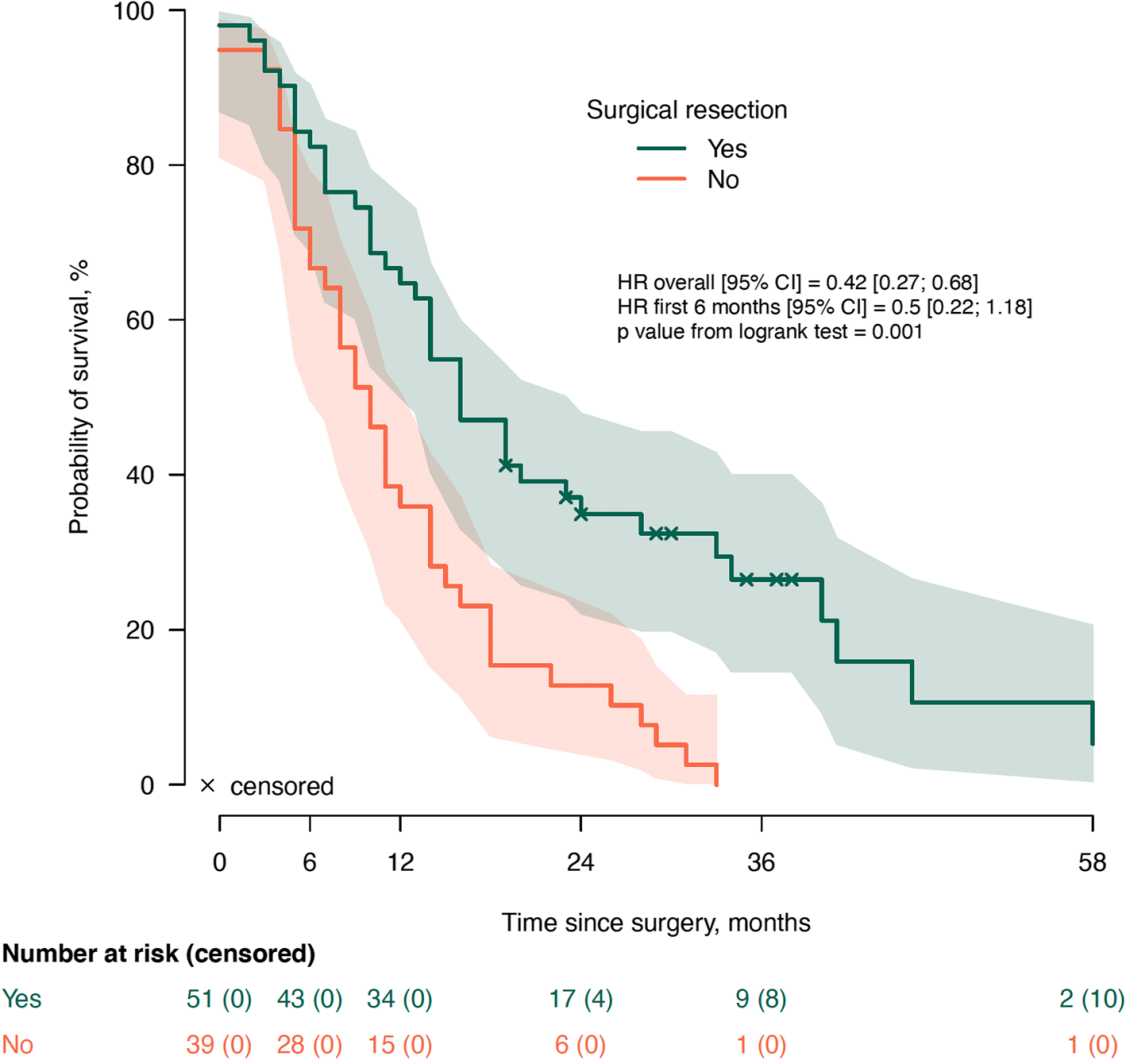
Overall survival according to surgical resectability

**Fig. 4:**
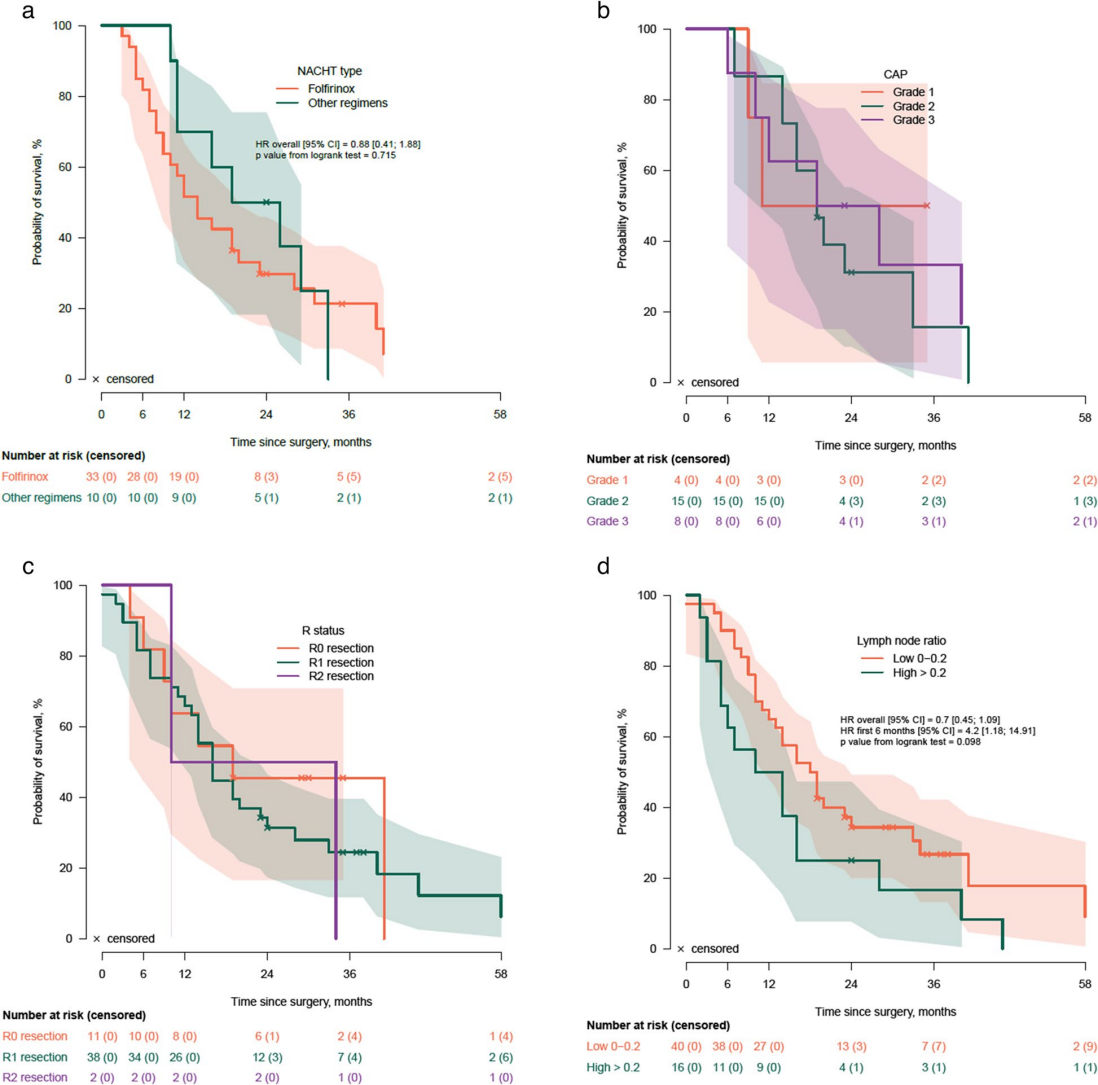
Stratified overall survival analysis according to: **a**, Type of neoadjuvant chemotherapy (FOLFIRINOX versus other regimens); **b**, Tumor response grading system of the College of American Pathologists (CAP) after neoadjuvant chemotherapy (0: no viable cancer cells; 1: single cells or rare small groups of cancer cells; 2: residual cancer with evident tumor regression, but more than single cells or rare small groups of cancer cells; 3: extensive residual cancer with no evident tumor regression); **c**, R-status; **d**, Lymph node ratio

Median follow-up since surgical exploration was 14 months (IQR: 7-23), during which 79/90 patients deceased (88%). The median OS times for both groups, BR (95% CI 11-17) and LA (95% CI 9-19), were 14 months, with no statistically significant difference (*p=*0.567) (Fig. 1). The median OS since surgery was 16 months (95% CI 11-21) in the group which received NACHT and 10 months (95% CI 5-15) in the group with upfront surgery (*p=*0.104) (Fig. 2). There was no significant difference in OS between the 33 patients who received FOLFIRINOX and [14 (95% CI 14-28)] and the 10 patients who received other regimens [19 (95% CI 16-28), *p=*0.733] (Fig. 4a). Twenty-seven patients underwent a surgical resection after NACHT. There was no patient with a complete pathologic response after NACHT (grade 0), 15% (4/27) had a grade 1 response, 55% (15/27) had a grade 2 response and 30% (8/27) showed a grade 3 tumor response on pathological evaluation. Survival analysis according to the grading of tumor response after NACHT did not show any significant differences in survival (*p=*0.791) (Fig. 4b). The median overall survival was 16 months (95% CI 12-20) in the group which underwent surgical resection and 10 months (95% CI 7-13) in the group with an unresectable tumor (*p=*0.001) (Fig. 3). Stratification analysis according to R-status showed no significant differences in OS between R0 (median 19, 95% CI 14-34) and R1 (median 16, 95% CI 17-30) resections (*p=*0.836) (Fig. 4c). Also, the OS for patients with a low LNR did not show a significant difference compared to patients with a high LNR [18 months (95% CI 19-33) vs. 10 months (95% CI 9-23), *p=*0.078] (Fig. 4d).

Using a simple Cox proportional hazards model, the mortality hazard was estimated to be 70% [39 - 85%] and 51% [19 - 70%] lower in patients with surgical resection without and with involved lymph nodes, respectively, compared to patients without surgical resection. These associations remained significant in a multiple Cox proportional hazards model considering NCCN classification and whether neoadjuvant chemotherapy was administered (and if so whether there was response) (Table 3). The multiple Cox proportional hazard from Table 3 showed no significant associations between the use of NACHT (with or without response) and mortality hazard. Likewise, we could not find a significant association between NCCN classification and OS (cHR 1.01; 95% CI 0.63-1.62; *p=*0.97) (Table 3).

**Table 3:** Cox proportional hazards analysis

**Variables**	**Simple analysis**		**Multiple analysis**	
	**HR (95% CI)**	***p*-value**	**cHR (95% CI)**	***p*-value**
**Surgical resection and if so involved lymph nodes**				
Surgical resection with NO involved lymph nodes vs No surgical resection	0.30 (0.15 - 0.61)	0.001	0.30 (0.14 - 0.65)	0.002
Surgical resection with involved lymph nodes vs Surgical resection with NO involved lymph nodes	1.65 (0.80 - 3.39)	0.172	1.62 (0.77 - 3.42)	0.206
Surgical resection with involved lymph nodes vs No surgical resection	0.49 (0.30 - 0.81)	0.005	0.49 (0.29 - 0.82)	0.007
**Neoadjuvant chemotherapy administered and if so RECIST**				
NACHT with stable disease or progression vs No NACHT	0.63 (0.31 - 1.26)	0.191	0.66 (0.33 - 1.33)	0.249
NACHT with stable disease or progression vs NACHT with response	0.86 (0.41 - 1.79)	0.682	0.71 (0.34 - 1.49)	0.365
NACHT with response vs No NACHT	0.73 (0.45 - 1.19)	0.213	0.94 (0.55 - 1.59)	0.804
**NCCN classification (LA vs BR)**	1.13 (0.73 - 1.76)	0.583	1.01 (0.63 - 1.62)	0.976

*RECIST* Response evaluation criteria in solid tumors, *NACHT* Neoadjuvant chemotherapy, *NCCN* National Comprehensive Cancer Network, *LA* Locally advanced pancreatic ductal adenocarcinoma, *BR* Borderline resectable pancreatic ductal adenocarcinoma

## Discussion

The treatment of BR and LA has been evolving with a wider application of multimodal treatment, in which perioperative chemotherapy is combined with surgery [2]. Surgical selection, feasibility and safety are primordial as futile R2 resections or complications can preclude patients from receiving chemotherapy and compromise their prognosis.

Our results show no difference in OS between BR and LA PDAC and surgical resection was the only factor associated with a longer OS, using both simple and multiple Cox proportional hazards models. In the BR group, the R1 resection rate was higher (54.5%) and the rate of unresectable tumors was lower (29.5%) in comparison to the LA group, with an inverse relation of lower R1 resection rate of 31.1% but higher rate of unresectable tumors (55.6%). The therapeutical decision was agreed upon during the tumor board meeting and our preferred strategy for LA involved NACHT. However, 18 patients with LA had upfront surgery because of one or more of these three reasons: presence of limited disease although categorized as LA; concerns about the patient's ability to tolerate a substantial dose of NACHT; patient's preference for immediate surgery. Tumor unresectability was in 85% of the patients of both cohorts due to local vascular infiltration, without systemic metastases in peritoneum or liver. Therefore, the difference observed in survival between the resected patients and the patients with an unresectable tumor, irrespective of NCCN-classification, is more likely attributable to the impact of successful tumor resection rather than the burden of systemic disease [2]. The study underscores that surgery is the sole factor associated with prolonged survival, and this positive difference becomes more pronounced over time.

Of the 51 patients who had a surgical resection (51/90, 57%), 6 patients with BR (6/31, 19%) and 5 with LA (5/20, 25%) had an R0 resection (Table 1). In the study population we performed no arterial resection. Of the 12 patients with pancreatic body LA, there were four (33.3%) R1 resections, no R0 resection and 8 (66.7%) patients were explored but did not undergo resection. Our technique involves periadventitial dissection with arterial divestment and if the frozen section of these tissues shows no tumor infiltration, then dissection would proceed. A high R1 resection rate in LA is not unexpected but we would not proceed to coeliac trunk resection unless there would be a clear invasion of the arterial wall during surgical exploration. This low R0 rate could be attributed to the standardized anatomopathological protocol applied with extensive sampling of the transection margins as well as dissection surfaces, with the 1 mm rule for a free resection margin [23]. It has been demonstrated that the R1 resection rate can be as high as 85% when a standardized pathological examination is applied after surgery for tumors classified as resectable PDAC based on imaging [24, 25]. The value of surgery should be emphasized even if an R1 resection is achieved by 1-mm rule while trying to accomplish an R0 resection.

In the BR group, 10 patients (23%) underwent a venous resection while in the LA group only 2 patients (5%) had a venous resection. NACHT was more frequently applied in patients with LA (62.8%) than in BR (35.6%). The favorable tumoral effect of NACHT could possibly enhance the feasibility of a vein sparing approach. The lower median number of involved lymph nodes in LA could probably also be explained by the higher application of NACHT in this group in contrast to BR. These findings are in line with the study from Wijetunga et al. where patients with LA received more cycles of NACHT and at surgical resection they underwent less venous resections (65.4% vs 85.3%, *p=* 0.013) and showed less nodal involvement (42.3% vs 70%) than patients with BR [26].

In this study a median OS of 16 months in the group who underwent surgery appears less favorable in comparison to other literature. This can be explained by heterogeneity in defining “overall survival” in the literature. In our analysis, survival time was calculated from the date of surgery, while in other retrospective studies a date before surgery was used as reference, such as the date of diagnosis or date of start of NACHT [26–29]. The last leads to a longer duration of follow-up, longer OS result and a survival selection bias if not all the patients are included in the analysis from the moment of diagnosis or start NACHT. Patients who drop-out or die during NACHT before surgical exploration should be included in the analysis from the date of diagnosis, otherwise it can lead to a possible false positive effect of NACHT on survival.

As our study only included patients who underwent surgical exploration, patients undergoing NACHT would have a time advantage in comparison to those who are immediately operated on, if survival would be calculated from the date of diagnosis. Therefore, the date of surgery was used as reference and Cox regression analysis showed no association between the use of NACHT and OS (Fig. 2, Table 3). If we would use the date of diagnosis as reference, there would be a significant difference in survival curves between the use or not of NACHT determined by the log rank test (see Additional file 1). Studies using the date of diagnosis as reference but only including the patients who underwent surgery tended to show a positive effect of NACHT such as in the study from the Mayo Clinic in which an association was described between extended duration of NACHT for BR and LA with response and survival [28].

In our sample, median OS since surgical exploration for patients with BR and LA who had a surgical resection was 16 months (Fig. 3), but it would be 23 months if we would calculate it from the time of diagnosis. In the randomized controlled PREOPANC trial, median OS after neoadjuvant chemoradiotherapy with gemcitabine for BR and resectable pancreatic cancer was also 16 months, in line with our results, with an intention to treat analysis defined from the date of randomization and 14 months after immediate surgery [30]. The controlled trial from Seoul National University for BR comparing chemoradiation with gemcitabine with upfront surgery showed also median OS of 16 months for the entire study population [31]. Survival in this study was calculated from the start of chemoradiation or date of surgery according to the study group.

When selecting patients for these complex surgeries, one has to take into consideration that postoperative complications can preclude patients from receiving adjuvant therapy and compromise their prognosis. Our results show acceptable major morbidity rate and mortality rate, even when dealing with more advanced disease and challenging situations. Major morbidity rate (Clavien-Dindo grade III and IV) in this group was 7.8% with a 10% clinically relevant (Grade B and C) pancreatic fistula rate and 90-day mortality rate of 3.3%. In the study from Michelakos et al. the reported major morbidity rate (Clavien-Dindo ≥3) was 16.3% and 30-day postoperative mortality rate was 1.4% in 110 patients resected after neoadjuvant FOLFIRINOX [27]. In the study from the Mayo Clinic group, which analyzed predictive factors associated with operative morbidity, mortality, and survival outcomes in 194 patients with BR or LA undergoing total neoadjuvant therapy, there was a 36% major complication rate, and 90-day mortality of 6.7% (13 deaths) [28]. In contrast to other studies reporting arterial resections in 10-33% of patients, we did not perform any arterial resections in our study. This is noteworthy as arterial resections are associated with higher morbidity and mortality rates while the invasiveness of the procedure should be subdued and kept in balance with the oncological prognosis [2, 26, 28, 31, 32].

The relation between LA disease and tumor biology does not seem consistent as selected patients with LA can have the same OS as patients with BR. In this study, NCCN classification did not emerge as a predictive factor for survival, also challenging the prognostic impact of the NCCN classification [2]. The classification in BR and LA is based on imaging which has its limitations when evaluating evolution of tumoral vascular infiltration. As a result, in our study 22% of the patients preoperatively classified as BR (10/45) were considered inoperable due to vascular infiltration during surgical exploration and should in fact be classified as LA (Table 1). However, the NCCN classification remains a preoperative iconographic categorization. Are BR and LA really two different entities or should they be considered as a spectrum of the same disease? The role of surgery in the treatment of BR and LA PDAC should be stressed. The therapeutic goal is to be able to select the patients with a favorable tumor biology, to justify cumbersome surgery with prospects of an oncologic and survival benefit. The obvious necessity to find biomarkers to assess the aggressivity of the tumor beyond anatomical landmarks and beyond the conventional radiological findings is the instigator to start a prospective study evaluating the role of radiomics, genomics and proteomics in the selection of patients with BR and LA after neoadjuvant FOLFIRINOX (PeRFormanCe Trial, NCT05298722) [33–37].

Limitations of our study are its retrospective and single center character involving a period of 10 years. The small sample size is also a limitation, in addition to the fact that the patients who progressed during NACHT and were not referred for surgical exploration could not be included in this analysis, which reflects a particular study population with inherent selection bias. Longer oncological follow-up was limited since many patients received further postoperative treatment in their initial referral institutions, which imposed a restricted access to detailed information.

## Conclusions

In our experience, only surgical resection was associated with significantly longer survival of patients with BR and LA PDAC, which was comparable and independent of their classification in BR or LA. Surgical resection should therefore be encouraged in LA PDAC even if it could result in a R1 resection. Prospective studies including patients from the moment of diagnosis are required to identify biologic and molecular markers which may allow a better selection of patients who will benefit from surgery.

### Supplementary Information

**Additional file 1.** Overall survival according to the administration of neoadjuvant chemotherapy calculated from the time of diagnosis instead of from the time of surgery.

## Data Availability

Data is available upon request to the corresponding author.

## Abbreviations

BR: Borderline resectable pancreatic ductal adenocarcinoma
LA: Locally advanced pancreatic ductal adenocarcinoma
PDAC: Pancreatic ductal adenocarcinoma
NCCN: National Comprehensive Cancer Network
OS: Overall survival
NACHT: Neoadjuvant chemotherapy
RECIST: Response evaluation criteria in solid tumors
CT: Computed Tomography
MRI: Magnetic resonance imaging
SMA: Superior mesenteric artery
SMV: Superior mesenteric vein
LNR: Lymph node ratio
IQR: Interquartile range
HR: Hazard ratio
cHRs: Conditional hazard ratios
