# Supplementary material for: Predictive factors for survival in borderline resectable and locally advanced pancreatic cancer: are these really two different entities?
Source: BMC Surg. 2023 Sep 30;23:296. doi: 10.1186/s12893-023-02200-6 (PMC10541717; doi:10.1186/s12893-023-02200-6)
Supplement: Supplementary file 1 — Additional file 1. Overall survival according to the administration of neoadjuvant chemotherapy calculated from the time of diagnosis instead of from the time of surgery. [file 12893_2023_2200_MOESM1_ESM.pdf]

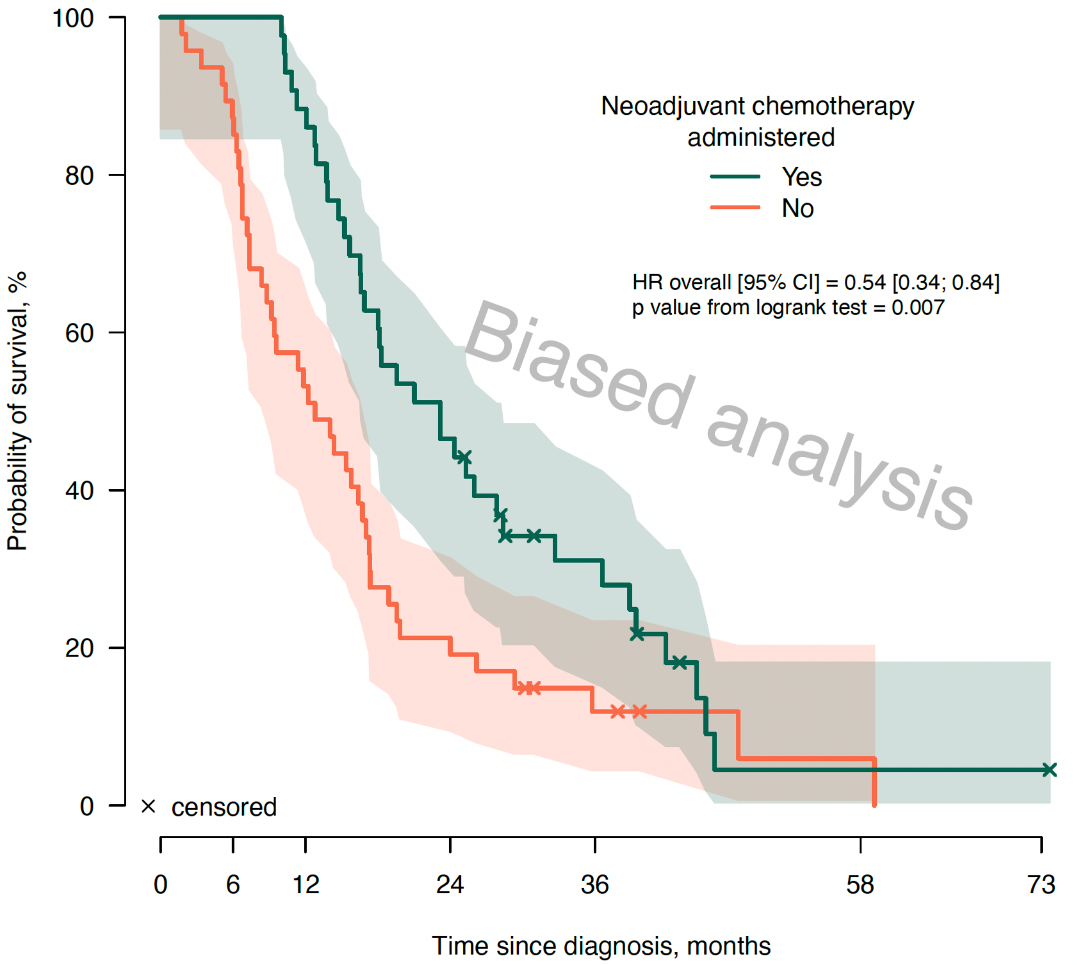

Number at risk (censored)

Yes	43 (0)	43 (0)	39 (0)	22 (0)	11 (4)	2 (6)	2 (6)
No	47 (0)	42 (0)	26 (0)	10 (0)	5 (2)	2 (4)	1 (4)

Additional file 1

Overall survival according to the administration of neoadjuvant chemotherapy calculated from the time of diagnosis instead of from the time of surgery.
